# Verteporfin Photodynamic Therapy Combined with Intravitreal Ranibizumab for Polypoidal Choroidal Vasculopathy Controversy Concerning Long-Term Followup

**DOI:** 10.1155/2012/897097

**Published:** 2012-10-30

**Authors:** Maribel Fernández, María Gil, Francisco Gomez-Ulla, Pablo Charlón

**Affiliations:** ^1^Department of Ophthalmology, University Hospital of Santiago de Compostela, Ramon Baltar S/N, 15706 Santiago de Compostela, Spain; ^2^Instituto Oftalmológico Gómez-Ulla, Avenida de las Burgas 2, 15705 Santiago de Compostela, Spain

## Abstract

*Purpose*. To show the long-term results of intravitreal ranibizumab combined with photodynamic therapy (PDT) for the treatment of polypoidal choroidal vasculopathy (PCV). *Methods*. We analyzed the progress of two patients for 36 and 58 months, respectively. We only used PDT for the treatment in the area of the active PCV or “hot spot” evident on the indocyanine green angiography (ICGA). The spot size was chosen so as to cover only the active neovascular lesion. We combined intravitreal ranibizumab with PDT when PCV remained active without visible polyps in ICGA or without a response to PDT. *Conclusion*. Administration, as required, of verteporfin photodynamic therapy combined with intravitreal ranibizumab is an effective treatment for symptomatic polypoidal choroidal vasculopathy. These data need to be confirmed in large, prospective, and controlled clinical trials which are randomized and carried out over a long period.

## 1. Introduction

Age-related macular degeneration (AMD) is the main cause of visual loss in the elderly population in industrial countries [1]. Exudative AMD is subcategorized into three phenotypes: typical AMD, polypoidal choroidal vasculopathy (PCV), and retinal angiomatous proliferation [1] but doubt still exists as to whether PVC is a distinct entity or a variant of neovascular AMD and other neovascularized maculopathies [2].

PCV is characterized by choroidal vascular networks with polyp-like aneurysmal dilations which are most clearly identified by indocyanine green angiography (ICGA) [3]. Fluorescein angiography (FA) and optical coherence tomography (OCT) are useful diagnostic tools [4]. In OCT, PCV is characterized by a higher incidence of retinal pigment epithelial detachment (PED), subretinal fluid, and less intraretinal fluid than eyes with exudative AMD [4]. PCV lesions appear as occult or minimally classic choroidal neovascularization (CNV) with FA, while ICGA clearly demonstrates the poly-like vascular network within the choroid. ICGA is the gold standard for the ultimate confirmation of PCV and it shows that the abnormal vasculature consists of a branching vascular network (BVN) and polypoidal lesions. Therefore, ICGA is the best way to diagnose PCV and to identify active components [5].

The visual prognosis of PVC has been reported to be better than that of exudative AMD, and a conservative approach is recommended, unless the lesion is associated with persistent or progressive exudative change that threatens central vision [6]. However, subretinal fibrosis and RPE atrophy can cause significant and permanent vision loss [7]. Moreover, the incidence of sub-RPE hemorrhage or subretinal hemorrhage in patients with PVC is high (30%–64%) [8, 9]. Controversy continues over the best way to manage active forms.

Among the currently available treatment modalities for PCV, verteporfin photodynamic therapy (PDT) seems the most promising [10], with complete regression of polyps in 73% to 99% of the patients and stabilizing or improving visual acuity (81%–100%) with typically fewer than 3 treatments [11, 12]. After PDT, the polyps are occluded, and exudative lesions are resolved [13–15]. Numerous reports describe the effectiveness of PDT for occluding polypoidal lesions and resolving the associated exudative changes [16]. However, PDT does not occlude the BVN. Future recurrence is inevitable as new polyps form from the remaining BVN [14]. Furthermore, a persistent BVN may leak in some cases, or PDT may induce the evolution of the BVN into CNV [14–16].

The polyps tend to recur, requiring frequent retreatment [17], and in these patients, PDT can cause serious complications, including massive macular hemorrhage, ischemia, or atrophy. Choroidal ischemia consecutively induces a secondary angiogenic response with increased expression of vascular endothelial growth factor (VEGF) [17, 18].

Anti-VEGF therapy is another treatment modality that is being investigated in PCV. Data available so far has indicated that anti-VEGF agents alone may not be effective for complete regression of polyps [7].

Compared with choroidal neovascularization seen in AMD, the vascular changes associated with PCV are more structured and mature, and this might explain their limited response to standard anti-VEGF therapy [17].

To achieve complete regression of the polypoidal lesions, while maximizing the visual outcome, combination therapy using PDT and anti-VEGF agents may be useful, rather than performing anti-VEGF monotherapy alone, as the former can combine the actions of angioocclusion and antiangiogenesis.

Ranibizumab is a humanized, antigen-binding fragment (Fab) with low molecular weight (48 kDa) that targets all VEGF-A isoforms and their biologically active degradation products [15]. Ranibizumab has a lower molecular weight and increased binding affinity for all forms of VEGF compared with bevacizumab [15, 16, 19]. Therefore, it is assumed to be more effective for treating PCV than is bevacizumab. Our choice of ranibizumab for intravitreal therapy was motivated by the fact that it is a relatively small molecule, which is more likely to reach the subretinal space where the polyps are located.

We analyzed the long-term results in two patients with PVC who underwent PDT and intravitreal ranibizumab. We reviewed the visual acuity, OCT, FA, and ICGA changes.

## 2. Materials and Methods

A retrospective review was performed to examine the medical records and angiographic data of 2 patients who were diagnosed with active PVC at the Gomez-Ulla Institute of Ophthalmology and the Department of Ophthalmology, Santiago de Compostela University Hospital. These patients were treated, as required, with PDT and intravitreal ranibizumab injections during 36 and 58 months of followup, respectively. The risk and benefits of PDT using verteporfin and alternative methods of treatment were described to the patients, who gave their consent to treatment.

Pertinent history, including age, sex, race, medical history, and previous treatments as well as clinical parameters (diagnosis, duration of macular exudation), was recorded. 

The diagnosis of PCV was based on the finding of characteristic poly-like choroidal vessel dilation, with or without a BVN, on ICGA using a confocal scanning laser ophthalmoscope (Heidelberg Retina Angiograph 2 (HRA2); Heidelberg Engineering, Heidelberg, Germany) or Topcon-IMAGEnet (Topcon corporation, Tokyo, Japan). Best-corrected visual acuity (BCVA), funduscopy, OCT, fundus photography, FA, and ICGA were performed on each patient before treatment. At each visit, the patients underwent a comprehensive ophthalmologic examination including BCVA, intraocular pressure measurement, indirect ophthalmoscopy, slip-lamp biomicroscopy with a contact lens, fundus photography, and OCT. Additional angiographies were performed as necessary.

BCVA was measured using Snellen charts. Firstly we used the stratus OCT (Carl Zeiss Meditec Inc., Dublin, CA), and then cirrus HD-OCT (Carl Zeiss Meditec Inc., Dublin, CA) was used at the baseline examination and at the follow-up visits. FA and fundus photography were performed on each patient using a Heidelberg Retinal Angiograph-2 (HRA-2; Heidelberg Engineering GmBH, Dossenheim, Germany) or a Topcon IMAGEnet (Topcon corporation, Tokyo, Japan).

The intravitreal administration of 0.5 mgr/0.05 mL of ranibizumab (Lucentis; Novartis International AG, Basel, Switzerland) was performed 3 times at 1-month intervals, and a standard PDT was performed using 689 nm diode laser system (Coherent Inc., Santa Clara, CA) after intravenous injection of verteporfin (Visudyne; Novartis AG, Basel, Switzerland). PDT with verteporfin was applied using the same parameters as for the treatment of neovascular AMD in the treatment of age-related macular degeneration with photodynamic therapy study [20].

Greatest linear dimension (GLD) was calculated based on ICGA, covering dilated polyps. Only the area of the active PCV or “hot spot” evident on the angiogram was treated. A spot size which would cover the active neovascular lesion was chosen. Retreatment was performed when ICGA showed active polypoidal lesions. The intravitreal administration of ranibizumab was considered when OCT showed persistent or new exudative change, including intraretinal edema or subretinal fluid in PVC with polyp lesions which were unresponsive to the treatments, or without polyps using ICGA, or when the polypoidal lesions persisted after treatments. 

The treatment end point was achieved when there was a complete absence of exudative change on OCT.

## 3. Case Reports

### 3.1. Case  1

**Figure 1:**
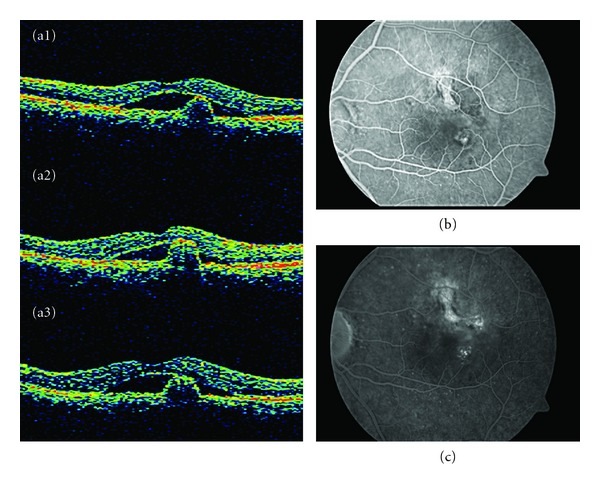
Stratus OCT shows a subretinal lesion with steep dome-like elevation suggestive of an active polyp with associated subfoveal neurosensory detachment (subretinal fluid). The patient was treated with three monthly intravitreal injections of ranibizumab with persistent hanges in the stratus OCT (A1, A2, A3). (b) and (c) The fluorescein angiograma (early and late phases) showed the nodular regions of hyperfluorescence associated with leakage as occult CNV. There was only staining of the ancient scar with minimal leakage.

**Figure 2:**
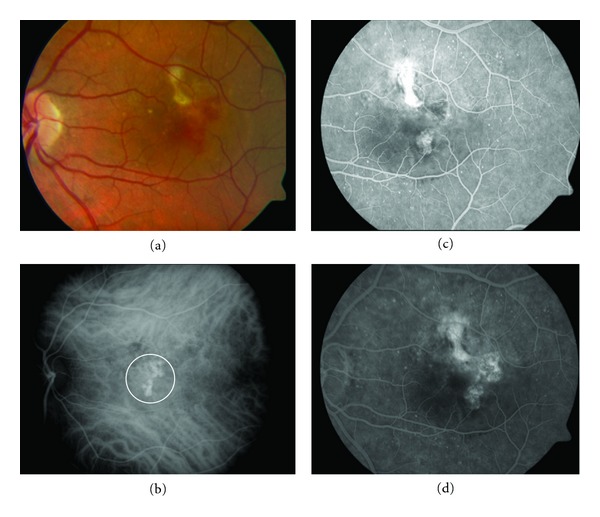
(a) Color photograph of the left eye, 6 months after treatment with three monthly intravitreal injections of ranibizumab, showing subfoveal neurosensory detachment with macular subretinal hemorrhage. (c) and (d) The fluorescein angiograma shows the same nodular regions of hyperfluorescence associated with leakage as occult CNV. (b) ICGA image demonstrating typical hyperfluorescent polypoidal lesions at the fovea (white circle).

A 54-year-old man attended a consultation regarding reduction of visual acuity (VA) in the left eye. He had received treatment with PDT for the same eye three years previously and had been stable up to the time of the consultation. At baseline, the BCVA was 20/50. FA showed occult CNV, and stratus OCT displayed subretinal fluid and changes to the retinal pigment epithelial (RPE) in the level of the fovea (Figure 1). The patient was treated with three monthly intravitreal injections of ranibizumab. Six months later, the BCVA was 20/40, and the FA showed an occult NVC and persistent changes in the OCT. An ICGA was performed and it revealed characteristics of typical subfoveal polypoidal lesions, and so it was decided to start treatment with PDT (Figure 2).

**Figure 3:**
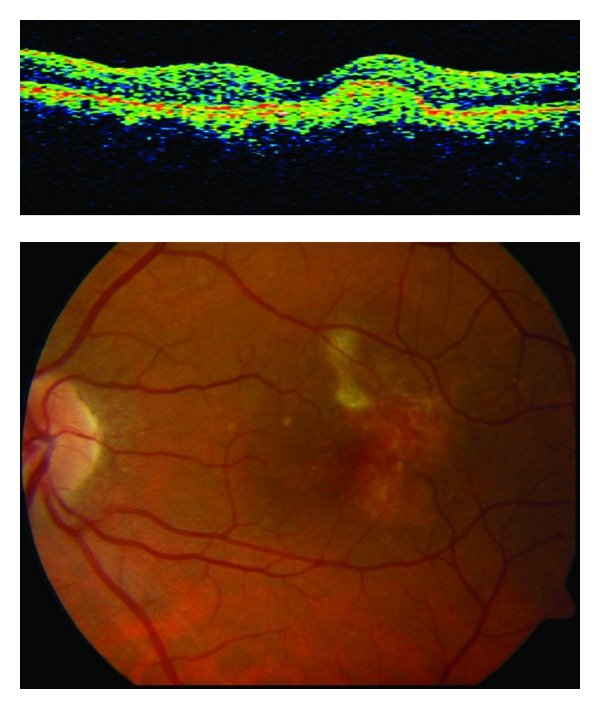
Three months later of the PDT treatment, the BCVA improved to 20/20 and stratus OCT did not show any exudative changes in the macula.

**Figure 4:**
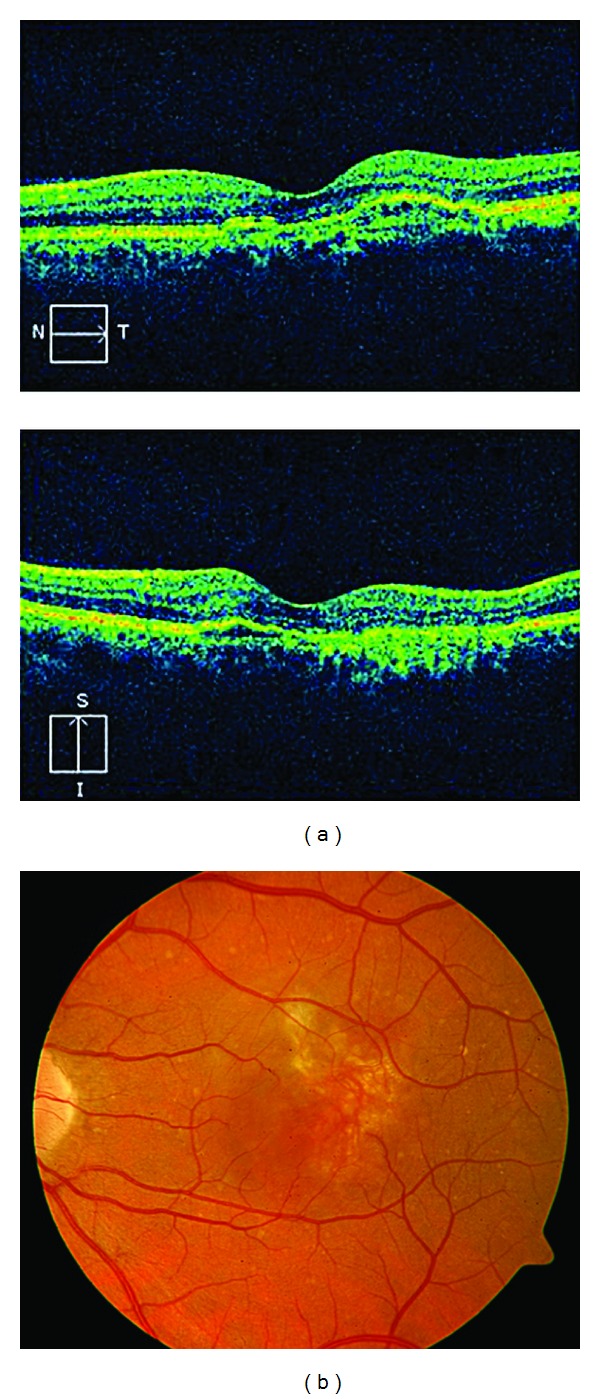
The patient remained stable for 42 months with a BCVA of 20/20 and without fluid in the stratus OCT.

**Figure 5:**
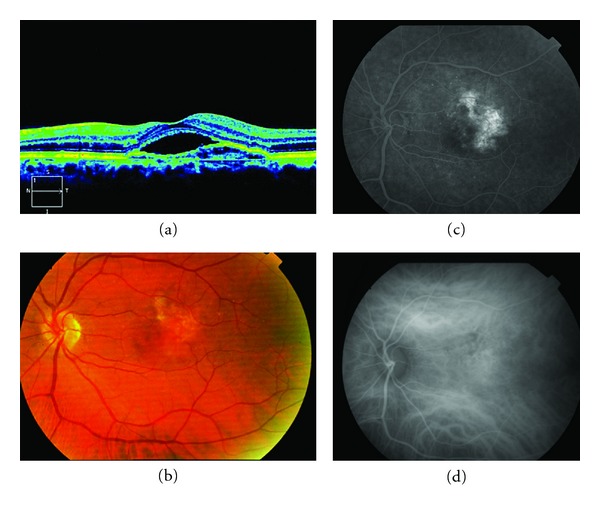
Forty-five months after the onset of symptoms, the patient reported a loss of visual acuity (20/30) and he presented a neurosensory detachment of the fovea in the cirrus HD-OCT (a), and it was therefore decided to repeat the FA and the ICGA. FA showed hyperfluorescence associated with leakage (c), and no polyps were observed in the ICGA (d), and so in view of the loss of VA and evidence of fluid in the cirrus HD-OCT (a), treatment of 3 monthly intravitreal injections of ranibizumab was carried out.

**Figure 6:**
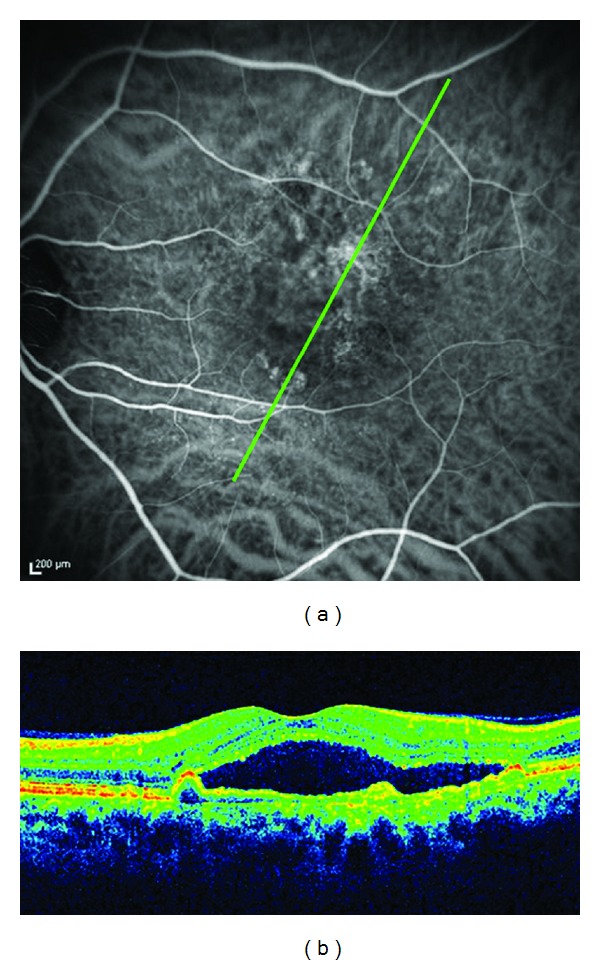
Six months after the unsuccessful treatment, a further angiography confirmed a recurrence of a new polypoidal choroidal vasculopathy in the eye. (a) ICGA showed new multiple polypoidal lesions at the extrafoveal area. (b) Cirrus HD-OCT confirms a subfoveal serous retinal detachment and a small protrusion of the retinal pigment epithelium corresponding to the polyp.

**Figure 7:**
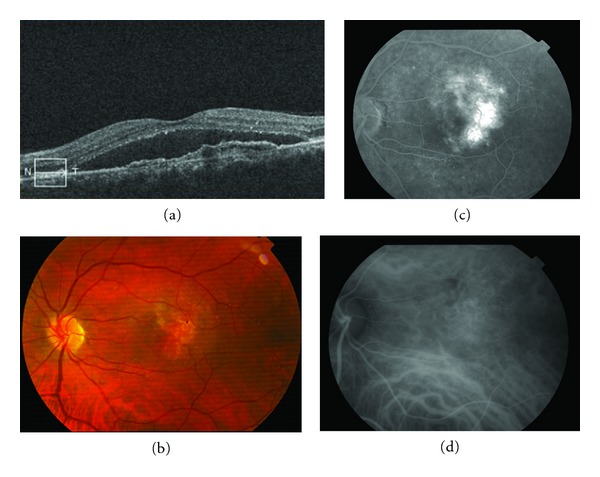
Three months after the second PDT, the polyps on the ICGA disappeared (d). In contrast, the exudative changes to the pigmentary epithelium of the retina in cirrus HD-OCT continued (a) and suggested the persistence of NVC ((a) and (b)).

PDT administration was guided by ICGA with a spot size of 2500 microns covering only the polyps (Figure 2(b)). Three months later, the BCVA was 20/20, and OCT did not show any exudative changes in the macula (Figure 3). The patient remained stable for 42 months with a BCVA of 20/20 and without fluid in the stratus OCT (Figure 4). Forty-five months after the onset of symptoms, the patient reported a loss of visual acuity (20/30) and he presented a neurosensory detachment of the fovea in the OCT, and it was therefore decided to repeat the FA and the ICGA. No polyps were observed in the ICGA, and so in view of the loss of VA and evidence of fluid in the cirrus HD-OCT (Figure 5), treatment of 3 monthly intravitreal injections of ranibizumab was carried out. However, visual acuity decreased to 20/50 in spite of the injections, and changes in the cirrus HD-OCT worsened rather than disappeared. Six months after the unsuccessful treatment, a further angiography confirmed a recurrence of polypoidal lesions in the eye (Figure 6), indicating the need for another PDT session on the polyps. Then, 3 months after the second PDT, the polyps on the ICGA disappeared. In contrast, the exudative changes to the pigmentary epithelium of the retina in cirrus HD-OCT continued and suggested the persistence of NVC (Figure 7). The AV was maintained at 20/40, and it was decided to monitor the patient progress given his poor response to the antiangiogenic treatment.

**Figure 8:**
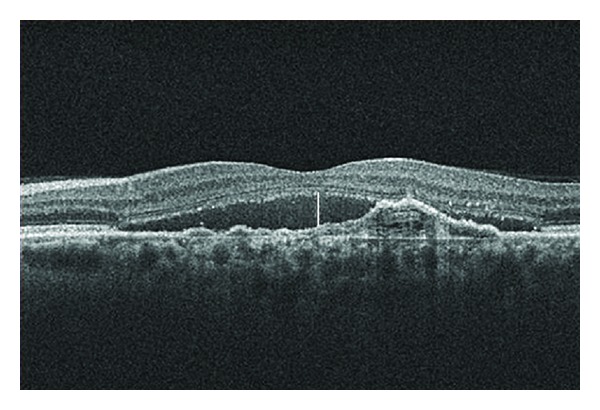
At 58 months of followup, the patient presented an BCVA of 20/30 and cirrus HD-OCT displayed typical changes of an occult CNV with a smaller quantity of subretinal fluid (white line), but persistence in the changes to the RPE.

At 58 months of followup, the patient presented a BCVA of 20/30, and cirrus HD-OCT displayed a smaller quantity of subretinal fluid, but with persistence of typical changes of an occult CNV to the RPE (Figure 8).

### 3.2. Case  2

A 65-year-old woman comes to urgencies for unilateral visual impairment and metamorphopsia in the right eye, dating from a month prior to her visit. The BCVA was 20/40, and at the fundus eye, a hemorrhagic detachment was observed with hard exudates at the macular level. She was referred to the Medical Retina Unit with a suspected diagnosis of PVC.

**Figure 9:**
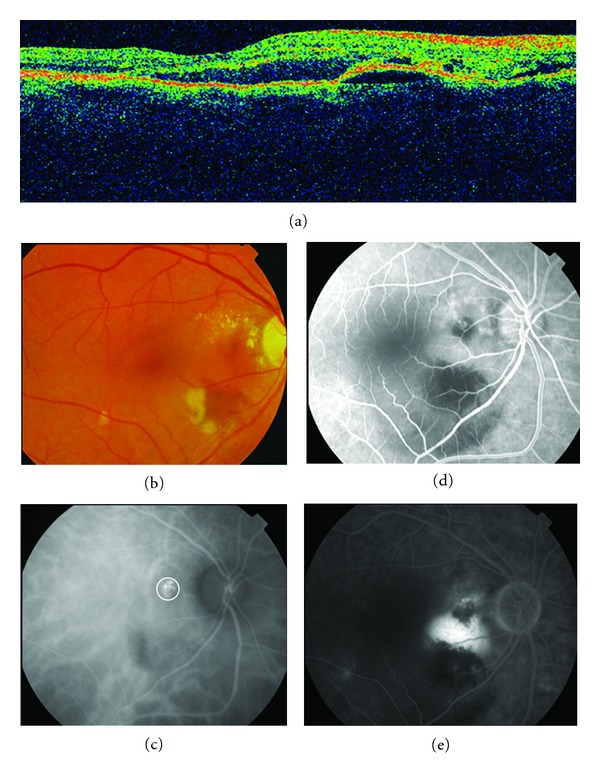
(a) Stratus OCT at baseline shows macular edema with subretinal fluid and retinal epithelial detachment. (b) Funduscopic photograph shows perilesional hard exudates and surrounding subretinal hemorrhage. (d) and (e) FA showed a pigment epithelial detachment (PED) with a subretinal hemorrhage. (c) Only a single juxtapapillary polypoidal lesion was observed with ICGA (circle).

**Figure 10:**
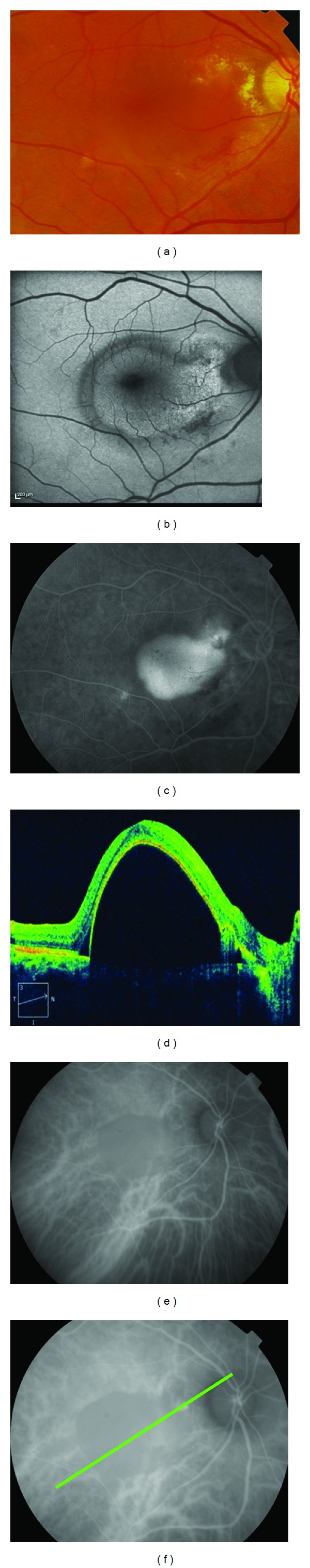
Three months later, the BCVA was 20/80, and although the condition of the fundus eye had improved (a), there was a great PED at FA and cirrus HD-OCT ((c) and (d)) with persistence of the polypoid lesion in the ICGA and cirrus HD-OCT ((f) and (d)), which led to a new PDT session on the polyp (spot size 1500 microns).

**Figure 11:**
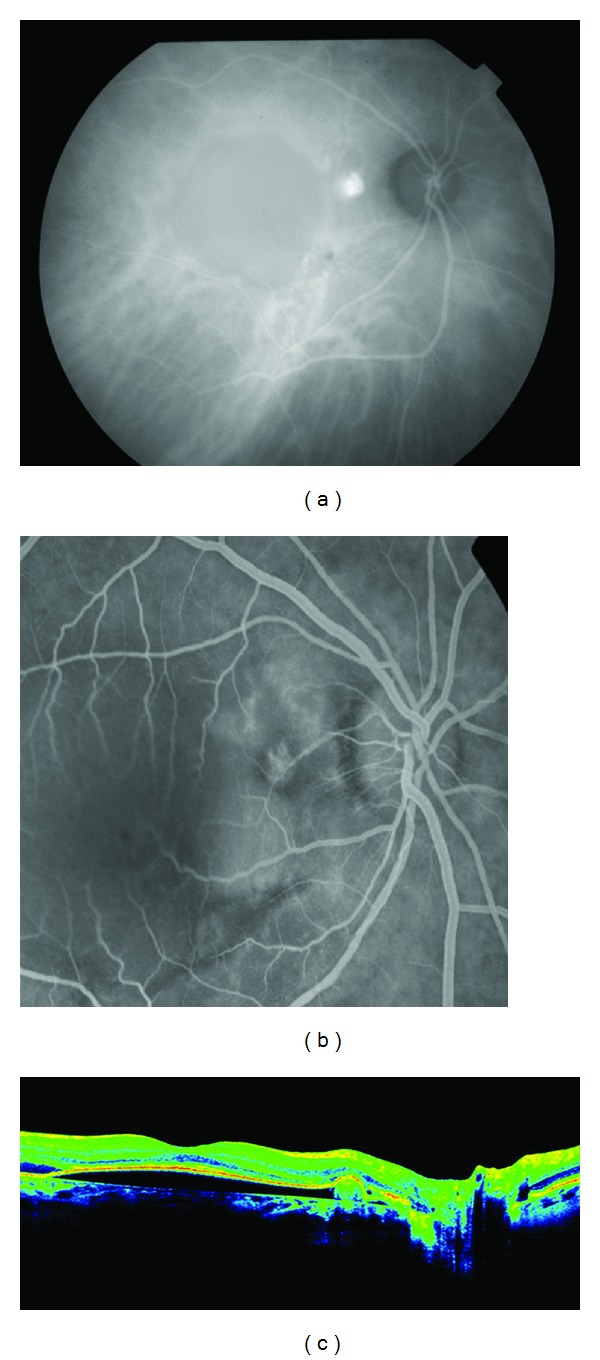
Three months afterwards, the PED still persisted in the ICGA and in cirrus HDOCT. FA, OCT scan, and ICGA show the persistence of the polypoidal lesion ((a), (b), and (c)), which resulted in the third session of PDT (spot size 1400 microns).

**Figure 12:**
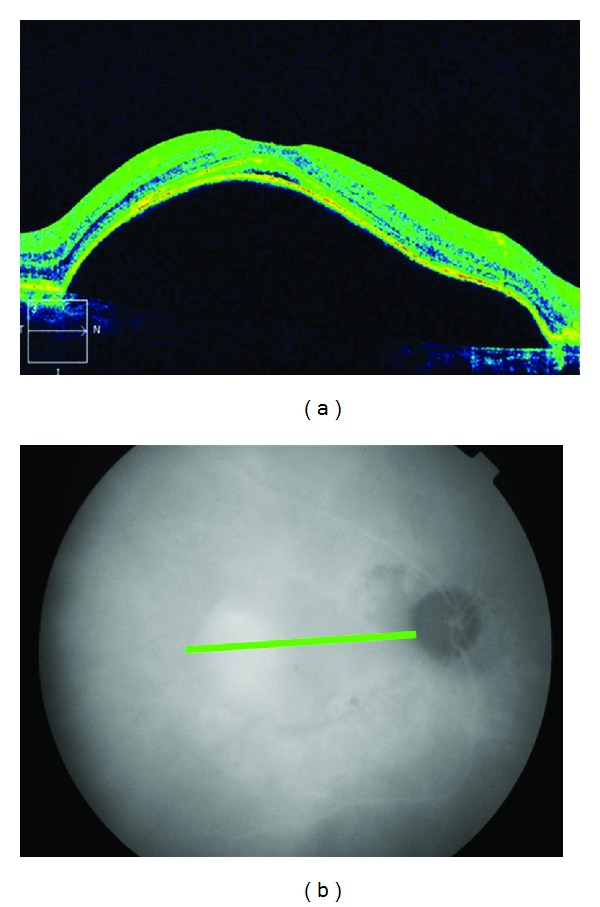
After a further 3 months, the BCVA was 20/50, and again, the PED persisted in the cirrus HD-OCT and in the ICGA, but polypoid lesions were not observed in either the cirrus HD-OCT or the ICGA ((a) and (b)).

FA showed a pigment epithelial detachment (PED) with a subretinal hemorrhage. Stratus OCT confirmed PED, and only a single juxtapapilary polypoidal lesion was observed with ICGA (Figure 9). We decided to perform a PDT session on the polyp (spot size 1100 microns). Three months later, the BCVA was 20/80, and although the condition of the fundus eye had improved, there was a great PED with persistence of the polypoid lesion in the ICGA, which led to a new PDT session on the polyp (spot size 1500 microns) (Figure 10). Three months afterwards, the PED still persisted in the FA, in cirrus HD-OCT, and the polyp in the ICGA, which resulted in the third session of PDT (spot size 1400 microns) (Figure 11). After a further 3 months, the BCVA was 20/50, and again the PED persisted in the cirrus HD-OCT and in the FA, but polypoid lesions were not observed in either the cirrus HD-OCT or the ICGA (Figure 12).

**Figure 13:**
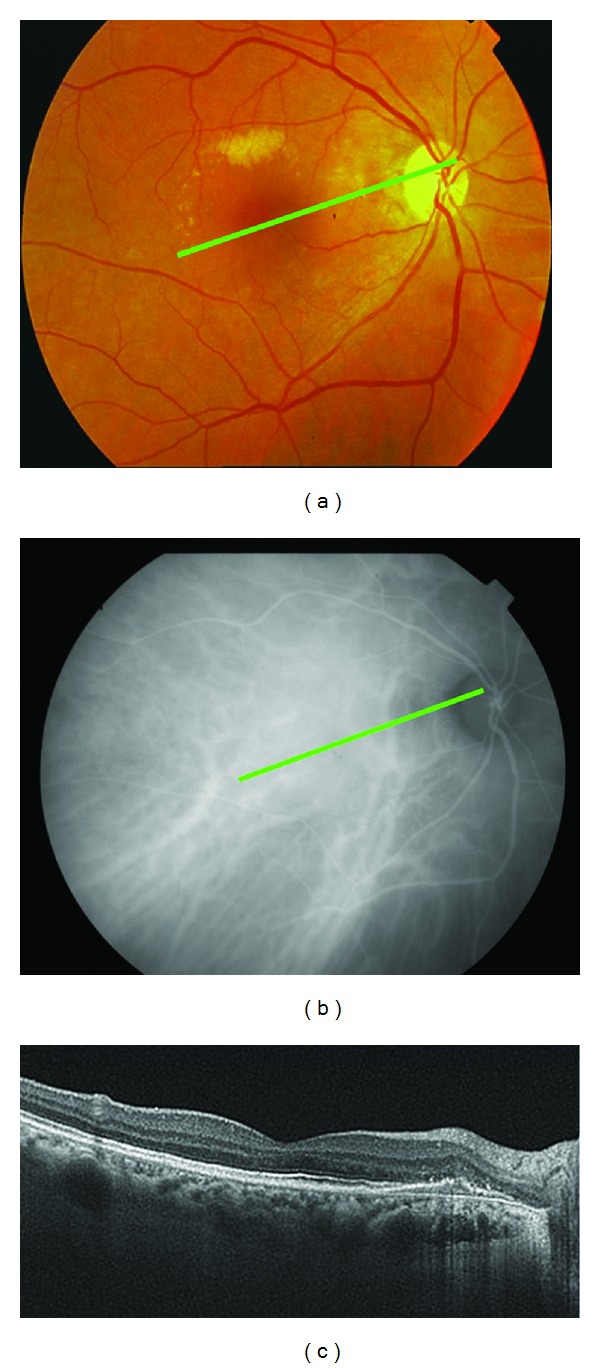
Six months later, after 3 PDT sessions on the single polypoid and 3 monthly intravitreal injections of ranibizumab, the PED was resolved, and the polyp disappeared ((b) and (c)). The patient remained stable with a visual acuity of 20/20 until month 36 of the followup.

Consequently, at 12 months of followup and after 3 PDT sessions on the single polypoid lesion, we decided the necessary treatment was 3 monthly intravitreal injections of ranibizumab, administered monthly. Six months later, the PED was resolved and the polyp disappeared. The patient remained stable with a visual acuity of 20/20 until month 36 of the followup (Figure 13).

## 4. Results and Discussion

Polypoidal choroidal vasculopathy is a clinical entity that is being diagnosed with increasing frequency because of improved imaging devices such as ICG angiography and OCT. Controversy continues over the way to manage active forms [18].

Photodynamic therapy with verteporfin has been the treatment of choice, especially in PVC [12, 14, 21–24]. Visual acuity is maintained or improved in 80% to 95% of patients. Some studies suggest that PDT resolves PCV lesions in less than 3 treatments [11, 12], but last studies with a long followup describes frequent recurrences after PDT [22, 25]. There is evidence that the PDT occludes the polyps but sometimes its tends to recur. The one major concern regarding the efficacy of PDT for PCV is the recurrence or the development of a new polypoidal lesion with longer followup. In some cases, the PDT cannot occlude the polyp properly but in other cases, the persistent branching vessels in the network may be origins of new active polypoidal lesions.

Periodical ICGA would be needed to detect abnormal choroidal vascular changes during long-term followup after PDT and in order to determine whether persistent or recurrent lesions are associated with polyps or BVN.

Although recurrence and retreatment do not seem to decrease BCVA further, some authors of previous reports agree on the need for studies with longer follow-up times to evaluate the safety and efficacy of PDT treatment of PCV [22]. PDT may alter the natural progression of the polypoidal CNV as a result of ischemia and increased VEGF expression [26]. After the introduction of anti-VEGF agents, several studies reported the efficacy of PDT with anti-VEGF treatment for PVC [27–29]as well as reporting on the way to minimize the complications related to the use of PDT and the need to use selective [5] or reduced fluence PDT [18].

Some studies suggest that high fluences could produce ischemia and stimulate VEGF production. Therefore, we speculate that the smaller spot or half the dose of fluence could be better because of reduced size. It is important because PDT may alter the natural progression of PCV in narrow sense, causing enlargement or a change to polypoidal CNV as a result of ischemia and increased VEGF expression.

In our study, the first patient did not initially respond well to ranibizumab on its own, but there appears to be a consensus that, in contrast to neovascular age-related macular degeneration, anti-VEGF monotherapy has shown limited effectiveness in patients with PVC [30]. Intravitreal anti-VEGF therapy has been shown to stabilize vision for a short period, reducing exudative changes, but it has shown little effect on choroidal vascular abnormalities [31, 32]. With a single PDT treatment, after the 3 intravitreal ranibizumab injections, the patient was stable for 42 months, with a BCVA of 20/20 and without fluid in the OCT image. We do not know if the antiangiogenic treatment associated with the PDT could have contributed to the positive results in the long-term followup in this case. The Everest Study showed that the disappearance rate of polypoidal lesions on ICGA was significantly better with PDT alone, and PDT plus ranibizumab, than with ranibizumab monotherapy [7].

It appears that the PDT on its own produces better results as regards occlusion of polypoid lesions. However, PDT plus ranibizumab could be useful for cases such as incomplete closure of active polypoid lesions, as in case 2, persistent exudative phenomena, or a finer branching vascular network. In these situations, the combination proved to be effective in preventing further visual deterioration [18]. Our patients maintained very good levels of visual acuity during a relatively long followup. We believe that the use of smaller spots could minimize the risks and side effects of PDT, and attempts to minimize these risks include a modified PDT protocol characterized by focusing the spot only on the polyps visible in ICGA, as if the PDT is not going to have an effect on the BVN [26, 33]. Moreover, the reduced number of PDTs might have been one of the reasons for the good visual outcome.When there are no visible polyps, the only option is treatment with anti-VEGF therapy, but if the patient does not respond, continuing does not appear to be justified. This was the case with patient 1, who appears to have a certain amount of resistance to ranibizumab.

We speculate that administration of VEGF antagonist for PVC, in conjunction with PDT, might minimize the adverse effects of PDT, as well as decrease the number of PDT treatments required [34]. During the long-term followup of our patients, we found a good visual acuity, perhaps due to the fact that the small size of the spot might produce less foveal atrophy and also due to the associated benefits of the anti VEGF treatment.

The combination of PDT and ranibizumab appears to be beneficial in prolonging the period before recurrence of polyps, as well as in maintaining good visual acuity [35]. The controversy continues, however, as we do not know the best way in which to combine them. Perhaps we need an individualized treatment, which will allow us to decide on the best choice to use them in each case. In our opinion, identification of PCV susceptibility genes represents a fundamental step to personalized therapy for this disease.

The use of combined PDT and intravitreal ranibizumab injections should be further evaluated in longer-term studies in order to determine if their synergistic effects will be useful in improving the long-term treatment outcome for PVC.

Our study was limited by its retrospective nature and by the number of patients.

## 5. Conclusions

Administration, as required, of verteporfin photodynamic therapy combined with intravitreal ranibizumab is an effective treatment for symptomatic polypoidal choroidal vasculopathy.

These data need to be confirmed in large, prospective, and controlled clinical trials which are randomized and carried out over a long period.
